# A Sentiment Analysis Approach to Predict an Individual’s Awareness of the Precautionary Procedures to Prevent COVID-19 Outbreaks in Saudi Arabia

**DOI:** 10.3390/ijerph18010218

**Published:** 2020-12-30

**Authors:** Sumayh S. Aljameel, Dina A. Alabbad, Norah A. Alzahrani, Shouq M. Alqarni, Fatimah A. Alamoudi, Lana M. Babili, Somiah K. Aljaafary, Fatima M. Alshamrani

**Affiliations:** Department of Computer Science, College of Computer Science and Information Technology, Imam Abdulrahman Bin Faisal University, Dammam 31441, Saudi Arabia; daalabbad@iau.edu.sa (D.A.A.); 2170005400@iau.edu.sa (N.A.A.); 2170006935@iau.edu.sa (S.M.A.); 2170007764@iau.edu.sa (F.A.A.); 2170007791@iau.edu.sa (L.M.B.); 2170004887@iau.edu.sa (S.K.A.); 2170006481@iau.edu.sa (F.M.A.)

**Keywords:** Arabic sentiment analysis, machine learning, support vector machine, K-nearest neighbor, naïve bayes, N-gram, natural language processing, Twitter

## Abstract

In March 2020, the World Health Organization (WHO) declared the outbreak of Coronavirus disease 2019 (COVID-19) as a pandemic, which affected all countries worldwide. During the outbreak, public sentiment analyses contributed valuable information toward making appropriate public health responses. This study aims to develop a model that predicts an individual’s awareness of the precautionary procedures in five main regions in Saudi Arabia. In this study, a dataset of Arabic COVID-19 related tweets was collected, which fell in the period of the curfew. The dataset was processed, based on several machine learning predictive models: Support Vector Machine (SVM), K-nearest neighbors (KNN), and Naïve Bayes (NB), along with the N-gram feature extraction technique. The results show that applying the SVM classifier along with bigram in Term Frequency–Inverse Document Frequency (TF-IDF) outperformed other models with an accuracy of 85%. The results of awareness prediction showed that the south region observed the highest level of awareness towards COVID-19 containment measures, whereas the middle region was the least. The proposed model can support the medical sectors and decision-makers to decide the appropriate procedures for each region based on their attitudes towards the pandemic.

## 1. Introduction

Coronavirus disease 2019 (COVID-19) is a novel disease reported in Wuhan China in December 2019. In March 2020, the World Health Organization (WHO) declared COVID-19 a pandemic, as the virus spread worldwide [1]. Countries around the world took measures to control the pandemic by encouraging people to follow precautionary measures recommended by health organizations, such as wearing facemasks and practicing social distancing. Most countries have imposed quarantines to slow the spread of the virus. Based on recent statistics, over 52 million people worldwide were affected by the coronavirus, with approximately 1.2 million cases of death [2]. This current situation has prompted people from all fields to provide quick solutions to contain the pandemic [3]. When it comes to quick spreading diseases, such as COVID-19, quick decisions need to be made regarding the practices the country needs to follow in order to control the disease, and its fast spread, such as imposing a full or partial curfew, converting to online education, locking-down airports, travel measures, etc. In this current crisis, the role of artificial intelligence (AI) has clearly contributed to medical decisions [3].

Sentiment analysis (SA), or opinion mining, is defined as the process of using machine learning (ML) and natural language processing (NLP) to classify emotions in subjective information. It is considered one of the most popular areas of research in the field of natural language processing, as it provides a means to review and analyze opinions that are held by any number of individuals [4,5]. In order to collect information related to a wide range of the population, social media platforms, such as Twitter, are valuable sources of such information. This platform has become very popular for individuals to freely express their feelings and share information on a daily basis towards any event.

The goal of this study is to collect and prepare a dataset that classifies an individual’s awareness in following precautionary measures during the quarantine period in the kingdom of Saudi Arabia, and then use the dataset to measure the awareness level of each region in the kingdom. The awareness will be identified based on the percentage of positive attitudes towards the precautionary measures expressed in an individual’s tweets in the targeted regions. The prediction of individual awareness will help medical sectors and the government to raise awareness, and make the right decisions (in the required procedures) to control the pandemic. The main contributions of this work are as follows: (1) to collect, preprocess, and prepare a dataset for Arabic sentiment analysis for COVID-19 precaution awareness in Saudi Arabia. The collecting and preprocessing steps discussed in this work can be applied to any Arabic tweet dataset. (2) Develop a machine learning model to classify and predict the individual’s awareness of the precautionary procedures based on tweets in the five regions of the Kingdom of Saudi Arabia. (3) To conduct a comparison between different machine learning techniques for classifying COVID-19 related tweets, and to select the best performing ones. (4) Finally, to apply the developed model, to predict the awareness of the individuals in Saudi Arabia’s five main regions, and to compare the level of awareness between these regions.

The rest of this paper is organized as follows: Section 2 discusses the related works. Section 3 presents the materials and methods. Section 4 presents the results and discussion. Finally, the conclusion and future works are identified in Section 5.

## 2. Related Works

Social media platforms, such as Twitter, are part of the dataset recourses that are widely used for sentiment analysis applications. The Twitter dataset provides a wide range of recent information related to user behavior, emotions, and opinions regarding events happening around the world [6]. Since the start of the pandemic, many researchers have studied and analyzed Twitter data by applying sentiment analysis techniques for different purposes. The work presented by Samuel et al. [7] aimed to conduct a textual analysis for United States tweets during the peak level of the COVID-19 spread, to identify the progress of fear sentiment that developed with the rapid spread of the virus and its associated consequences. The dataset was collected from Twitter based on crisis scenario keywords. Analysis was conducted by applying and comparing two machine learning techniques, which are Naïve Bayes and Logistic regression. The results showed that with short-length tweets, the Naïve Bayes (NB) classifier achieved high classification accuracy at 91% compared to the logistic regression, which scored 74% accuracy.

Other works focused on characterizing Twitter comments related to public opinions toward quarantines that were imposed around the world due to the pandemic. In research conducted by Anderson [8], 80,000 tweets were collected and analyzed to investigate the anti-quarantine comments expressed by individuals during that period in the United States. Six hashtags were used related to anti-quarantine expressions, such as: #End_the_Lockdown and #antiquarantine. Text mining techniques for the English language were applied for the analysis. The results showed that anti-quarantine topics could be summarized in 11 topics related to health issues, business, comparing COVID-19 with the flu, and other similar topics.

The impact of using social media during the pandemic was also studied in the work presented by Shi et al. [9], which illustrates the role of social bots in manipulating public opinion in social media, which illustrates the role of social bots in threatening the public health by manipulating the public opinion in social media. The study focused on the three major peak periods of the pandemic and conducted a sentiment analysis of Twitter data to analyze the opinions and expressions generated by social bots and compare them to those generated by humans. The findings showed that the use of sentiment polarity between the two is almost similar for all topics related to COVID-19. Moreover, it was discovered that social bots were used more for negative expressions regarding the situation.

Studying an individual’s emotions towards the COVID-19 pandemic in social media was discussed in [10,11]. The two studies focused on Spain and used the social media and ecosystem as a source of data to analyze the anger, fear, and sentiment of the public during the pandemic. The sentiment analysis included classifying different emotions, such as sadness, anxiety, happiness, etc., based on the tone. The studies explained the impact of the pandemic on emotional health.

Focusing on the Arabic language, natural language processing (generally) and sentiment analysis (especially) is a challenging task [12]. This challenge arises from several factors, including the morphological complexity of the language, the large number of forms and dialects, the diacritization, and the grapheme-to-phoneme conversion [13,14]. Due to these addressed challenges, the sentiment analysis process for Arabic needs to go through a number of preprocessing steps, which are discussed and applied in this work.

Despite the challenges, many works have been done for Arabic sentiment analysis. The focus of those studies mainly rely on data collection and preprocessing, besides training and testing several machine leaning classifiers with different datasets [15,16]. A wide range of studies targeted Arabic tweets for sentiment analysis, such as the study presented by Al-sukkar et al. [17], which used sentiment analysis to classify Arabic tweet polarity as positive or negative. They used a different combination of techniques for preprocessing, such as tokenization, filtering, stemming, and generating N-gram models to enhance the quality of the results. Next, they created a dataset of tweets where values of this dataset matrix were based on different schemas, such as Term Frequency–Inverse Document Frequency (TF-IDF), term frequency, and term occurrence. To classify the tweets, Support Vector Machine (SVM) and Naïve Bayes (NB) were applied using the 10-cross validation. The results showed that SVM scored the highest accuracy of 83.16% when using unigram without filtering stop words. NB came second with an accuracy of 81.93% by using 2-g/3-g and stop words filtering.

Alomari et al. [18] processed modern standard Arabic and Jordanian Arabic Twitter corpus of 1800 annotated tweets to achieve 88.72% accuracy, by applying SVM with TF-IDF weights combined with bigram stemming.

Several machine learning classifiers were applied in the work presented in Al-Tamimi et al. [19], which aimed at analyzing sentiments of 5986 Arabic YouTube comments, classified to positive and negative sentiments. SVM based Radial Basis Function (RBF), K-nearest neighbors (KNN), and Bernoulli NB models were used to classify raw and normalized datasets. The SVM-RBF model scored the highest accuracy of 88.8% once applied with normalized data.

In the study presented by Alhajji et al. [20], sentiment analysis of Arabic tweets was conducted by applying NB to run Arabic sentiment analysis of tweets through a Python library: Natural Language Toolkit (NLTK). They avoided laborious work by using a labeled dataset of Arabic tweets in which its sentiment labels are based on emoji lexicons. The dataset was split into 50% positive and 50% negative. A total of 47,000 tweets were used for training, and 11,000 were used for testing. The testing results acquired by using a unigram NB achieved 0.89 accuracy, 0.92 precision, 0.86 recall, and 0.89 F-score. The applied preprocessing techniques were stemming, normalization, tokenization, and stop words removal.

Behdenna et al. [21] used Arabic sentiment analysis to identify an opinion expressed in Arabic tweets. They collected 500 tweets and manually tagged them. First, tweets were extracted and tokenized into words and converted from text into Attribute-Relation File Format (ARFF) to be used by Weka tool 3. Second, 10-fold cross-validation method was used to train SVM and NB classifiers. The achieved results scored 82.1% for precision, and 81.4% for recall with the NB classifier. SVM scored the highest precision and recall at 86%.

In conclusion, sentiment analysis is proven to be a valuable source of information mining, especially for situations related to the need of analyzing a large amount of data related to the public, such as studying public behavior towards the COVID-19 pandemic, and its effect on public life. Moreover, it can be concluded that the three machine learning classifiers: SVM, NB, and KNN, are widely used in this field due to their high accuracy.

## 3. Materials and Methods

**Figure 1:**
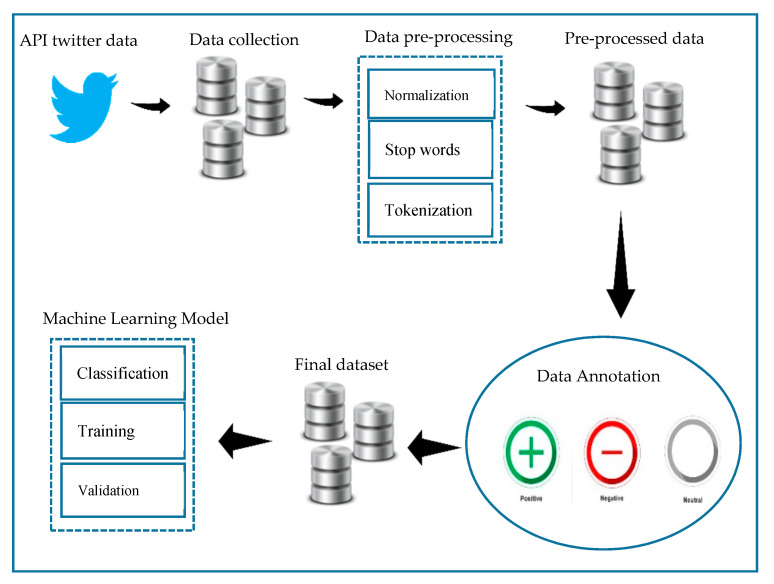
The framework for awareness prediction sentiment analysis approach.

In this section, the main components are discussed, including: data collection and preprocessing, main feature extraction using natural language processing techniques, and the development of several machine learning models using different classifiers. Figure 1 shows the framework of the proposed model.

### 3.1. Data Collection

Since the targeted period is the quarantine, the Twitter dataset was chosen to be the data source due to the fact that Twitter was used extensively by individuals to express their feeling towards the situation. The dataset was downloaded from Twitter using TWINT which is an advanced Twitter scrapping tool. The Kingdom of Saudi Arabia was divided into its five main regions (south, north, east, west, and middle), and then tweets were collected for each region based on location. As this study targeted Arabic tweets only, the most related Arabic keywords and hashtags to the scope were used, such as (كوفيد_19# كورونا# عدم_الخروج_من المنزل# ارتداء_الكمامة# التعقيم# التعليم_عن_بعد # غسل_اليدين#التباعد_الاجتماعي #) (#Covid #Corona #Stay_Home #Wearing_mask #Sterilization #Distance_learning #Washing_hands #Social_distance). These keywords represent the main precautionary procedures, such as COVID-19, corona, wearing masks, hand washing, social distancing, stay home, etc. Furthermore, only tweets that lie in the quarantine period in Saudi Arabia, which was from 23 March 2020 until 21 June 2020, were collected. The total number of collected tweets was 242,525. Duplicated and unrelated tweets were excluded.

### 3.2. Preprocessing

In this stage, preprocessing techniques were applied in order to clean the tweets by removing unwanted symbols. The main aim of this stage was to prepare the collected data to be used with the developed models, for training and validation. To achieve this goal, several steps were applied as follows.

#### 3.2.1. Normalization

Normalization is a preprocessing approach aimed at cleaning data, to have a sequence of texts with standard uniform [22]. For the collected data, all emojis, URLs, and special characters were removed. For the hashtags, two cases were identified. The first were tweets in which hashtags represented part of the sentence and completed the meaning. Those hashtags were kept in the tweets due to their roles in identifying the correct meaning. The second case included hashtags that were not considered part of the tweet, as they did not affect the understanding. These hashtags were removed.

Moreover, Taskhkeel (diacritics) and Tatweel (repeated letters) cases were handled in the normalization process, where all repeated characters, such as “غررررريب”, were converted to their correct form, e.g., “غريب”, for the previous example. In addition, Arabic characters that had multiple forms, such as Alef, which is written in multiple ways “ أ– إ - آ “, were all normalized to their single form “ا” for a unified representation.

#### 3.2.2. Stop Words Removal

Stop words are the words that do not affect the meaning of the sentence, such as conjunctions, prepositions, articles, and pronouns [23]. In this process, tweets were split and compared with the Arabic Stop Words library, which was created for this research purpose. The negation stop words, such as (‘ لا’, ‘غير’, ‘دون’, ‘ألا’,’الا’) (except–no-none-without-with), were kept in the sentence due to their importance in identifying the meaning of it.

#### 3.2.3. Tokenization

Tokenization is the process of converting the text into tokens separated by white space. In tokenization, all special characters are removed, the word boundaries are determined; abbreviations and numbers are processed [24]. For Arabic, the number of tokens may reach four tokens due to the morphological complexity of the language. Arabic words usually contain a number of affixes and clitics; hence, a segmentation process to remove suffixes was applied before conducting the tokenization process.

### 3.3. Annotation

Tweet annotation is the process of categorizing tweets based on the research scope [25]. The dataset was annotated into three major categories. These categories were related to the individual perception of the awareness of the precautionary procedures to prevent COVID-19 outbreaks in Saudi Arabia in terms of positive, negative, and neutral perception. A similar number of tweets for each category were annotated manually. The positive category included all tweets that showed awareness towards precaution measures and confirmed following them, such as wearing masks, social distancing, staying home, and/or washing hands, etc. On the other hand, the negative category included tweets that indicated carelessness and denying of COVID-19, and the required precautions, such as going out for no reason, not wearing masks or gloves, or visiting friends and family, etc. The neutral category contained tweets that included both positive and negative behaviors in the same tweet, for example: “I went out to buy coffee during quarantine with mask on“. The classification was based on specific indicators of keywords.

### 3.4. The Features Extraction

Different sentiment analysis models were built using different techniques, in order to identify which technique provides higher accuracy with the selected COVID-19 related twitter dataset for the Arabic language. The first step of the model development was the feature extraction process, aimed at finding a suitable set of features that improves the classification accuracy. For these tasks, different N-gram models were used. The second step of the experiment was to train the dataset with a number of machine learning classifiers that proved to provide high accuracy in sentiment analysis for similar cases. Three machine learning classifiers were used: Support Vector Machine, Naive Bayes, and K-Nearest Neighbor. Supervised classification was applied with three class labels for the data (positive, negative, and neutral). The experiments aimed to find the feature extraction-classifier combination that worked better for classifying the set or Arabic tweets related to the awareness of the COVID-19 pandemic, and then used the best model to classify the tweets in order to predict the level of awareness for individuals in each region.

#### 3.4.1. N-Gram Method

N-gram model is one of the most popular natural language processing models that is widely used. By comparing different NLP models, N-gram is proofed to provide the best performance among other used models [26]. N-gram, basically, is the count of n sequence of words. N could be one-word level (called unigram), two-word level (which is called bigram), or a three-word level (which is called trigram) [27]. In the current work, unigram and bigram models were applied.

#### 3.4.2. Term Frequency–Inverse Document Frequency (TF-IDF) Method

TF is the abbreviation for Term Frequency, which is the number of times the word appeared in the document. IDF is the abbreviation for Inverse Document Frequency, which reduces the weight of the word that occurs in different documents [27]. This method is preferred because it focuses on the most distinctive words in the text, which overcomes the limitation of depending on word counts. The mathematical functions are shown in Equations (1) and (2):

(1) $$\mathrm{tf}\left(t,d\right)=\log\left(1+f_{t,d}\right)$$

(2) $$\mathrm{idf}\left(t\right)=\log\left(\frac{1+N}{1+n_{t}}\right)$$

tf(t,d): is the count of term **t** in document **d.** N: total number of documents. n: number of documents that contains term **t**.

### 3.5. Machine Learning Model

The next step, after features extraction, was to build the machine learning model. The previously prepared dataset was used once with each of the three ML classifiers: SVM, NB, and KNN.

#### 3.5.1. Support Vector Machine Classifier

In the research conducted in [28], SVM classifiers were used to classify Arabic social media posts into positive and negative. The highest accuracy achieved was 89% by using the SVM. Based on the work presented in [23], which conducted a review of the major research works in Arabic sentiment analysis, which included more than 20 works, the SVM classifier proved to provide high accuracy. Therefore, it was applied in this research.

SVM is a supervised machine learning algorithm that is used in classification and regression. It could be used for unsupervised machine learning as well. It works by plotting the data values on the n-dimensional space (n = number of features). The classification process is done by finding the hyper-plan that best splits the two classes [29]. Kernel function in SVM is used to transform the data into the proposed form. In this research, the linear kernel SVM was used, as it is known for providing the best results [29]. Equation (3) shows the linear kernel SVM.

Linear Kernel: k(x, x′) = x^T^x′ (3)

#### 3.5.2. Naïve Bayes Classifier

Many researchers have experimented with Naïve Bayes for solving sentiment analysis problems. The experiments conducted in [30] applied three classifiers: KNN, SVM, and Naïve Bayes with different sentiment analysis strategies on two different datasets. The first set contained movie reviews, where the other was a political purpose dataset. The results showed that applying Naïve Bayes scored the highest accuracy of 96.6% with the first dataset, and 85.7% for the other set.

In another study discussed in [31], Naïve Bayes and KNN performance were evaluated on a dataset of movies and hotel reviews. The obtained results showed that using Naïve Bayes performed better than KNN with the movie dataset, while for the hotel dataset, both classifiers performed similarly.

Naïve Bayes is a supervised machine learning classifier based on the statistical method, used for classification problems, by finding the probabilities of attributes [32]. It has several uses and applications, such as diagnostic classifications, classifying texts and documents, sorting and identifying spam emails, and predictive models [19,27]. NB classifiers work by finding properties, assuming that features are independent of each other. For this work, the features used were the ones extracted using N-gram models, as explained previously. Equation (4) shows the NB model.

(4) $$P\left(C|X\right)=\;\frac{P\left(X|C\right)\;P\left(C\right)}{P\left(X\right)}$$

where P(C|X) is the posterior, P(X|C) is the likelihood, P(X) is the predictor prior probability, and P(C) is the class prior probability. Naïve Bayes classifier is known for its ability to work with a small training dataset, and is an easily implemented model [33,34]. Among the different implementations of the NB classifier, this work applies the Gaussian Naïve Bayes method that accepts continuous input.

#### 3.5.3. K-Nearest Neighbor Classifier

KNN is used widely for sentiment analysis purposes due to its ability to support multi-labels. It is also used for its simplicity and efficiency in classification [29,35].

K-nearest neighbors (KNN) is a supervised machine learning algorithm, known also as instance-based learning [29]. KNN works by calculating the distance between the target value and the rest of the values, then finds the closest k-nearest neighbor values, and uses it to vote for the label [36]. The KNN classifier needs two parameters to be set: the k value, and the distance metric. In this research, the Euclidean function was used for distance calculation. K value was set to 9.

## 4. Results and Discussion

Experiments were conducted using the prepared dataset that contained positive, negative, and neutral classes. Moreover, 80% of the dataset was used for training, where 20% of it was used for validation with the implemented combinations of the feature extraction-classifier models. The proposed method involved applying different N-gram models with a set of classifiers. Features were extracted using four N-gram models: unigram, unigram TF-IDF, bigram, and bigram TF-IDF. These four feature extraction techniques were applied once with each of the classifiers: SVM, NB, and KNN.

Table 1 shows the obtained results of the models. The SVM classifier achieved the highest accuracy performance compared to the other classifiers with all N-gram models, as the accuracy of the SVM was always above 81%. The performance measures included accuracy (the proportion of correctly classified test records), precision (positive predictive value), recall (negative predicted value), and f-score (the harmonic mean of precision and recall).

**Figure 2:**
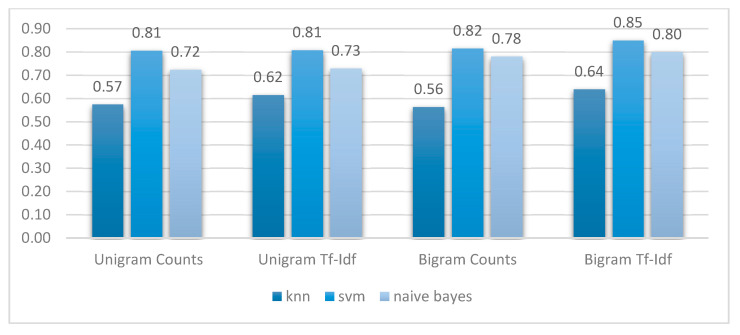
The accuracy using N-gram methods (unigram, unigram TF-IDF, bigram, bigram TF-IDF) with the classifiers (SVM, NB, KNN).

Figure 2 shows the accuracy results obtained from running the three classifiers (SVM, NB, KNN) with the different N-gram models. The obtained results illustrate that the bigram TF-IDF improves the performance for all classifiers compared to the other used N-gram models. Therefore, it is the best feature extraction method to be used for this research purpose.

**Figure 3:**
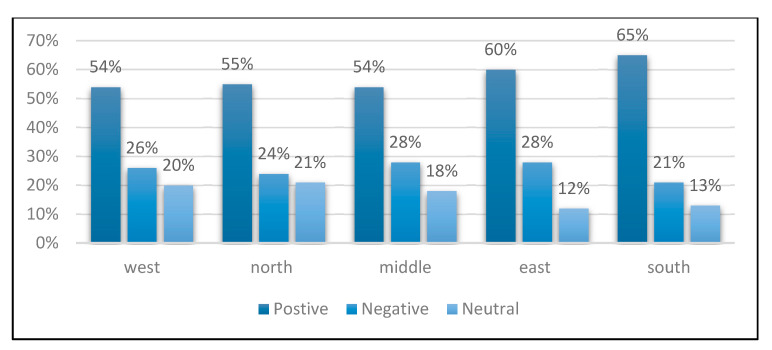
The percentage of each class for Saudi Arabia five regions.

By comparing the performance of the three classifiers with the bigram TF-IDF, SVM obtained the highest accuracy at 85%, and scored 87%, 84%, and 82% for precision, F-score, and recall, respectively. The least accurate performance was obtained with the KNN, which scored 64%. Therefore, the best-performed model (SVM with bigram TF-IDF) was used to classify the collected tweets dataset into the polarity that matches its content. The percentage of each class for each region was calculated, the results are displayed in Figure 3.

The percentage of the positive and negative tweets was used as an indicator of the increase or the decrease in awareness level towards the precautionary measures imposed to encounter the COVID-19 pandemic.

The results revealed that the south region of the country showed the highest awareness level as it scored the highest percentage of positive tweets of 65%. On the other hand, the middle and west regions had the lowest percentage of positive tweets, with 54%. The east region came second (in terms of awareness level) with 60% positive tweets, and then the north region with 55% positive tweets.

The high percentage of negative tweets was interpreted as carelessness towards the precautionary measures. The south and north regions scored the lowest percentage at 21% and 24%, respectively, which made them highly aware regions. Whereas the middle and east regions showed the highest percentage of negative attitudes towards the pandemic at 28%. The west region was ranked as the second least aware region as it scored 26% negative attitudes.

Neutral tweets indicate both negative and positive attitudes. The east region had the lowest percentages of neutral tweets with 12%, followed by the south with 13%. The north and west regions had the highest percentages, with 21% and 20% neutral attitudes.

**Figure 4:**
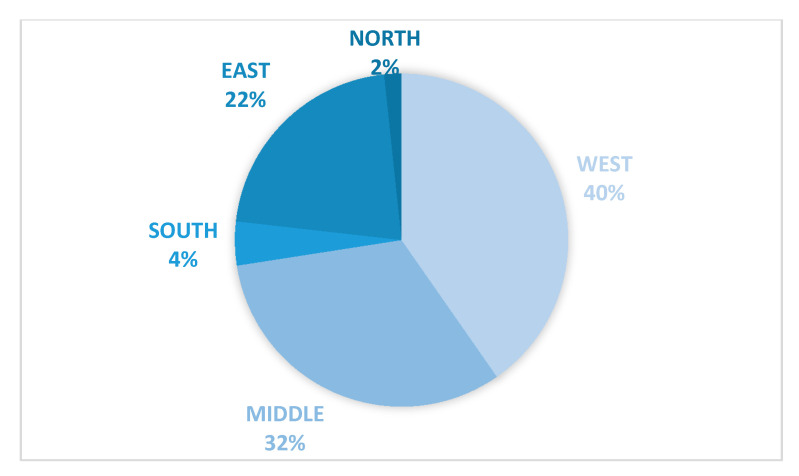
The total number of coronavirus disease 2019 (COVID-19) confirmed cases per region.

The results revealed that the individuals in the south region showed the highest level of awareness and responsibility towards following the recommended precautionary measures to slow the spread of the coronavirus. On the other hand, middle region individuals showed a lower percentage of awareness compared to other regions of Saudi Arabia. To justify the results, the total number of COVID-19 confirmed cases in Saudi Arabia, during the same period of the study, were investigated [34]. Figure 4 shows the percentage of COVID-19 confirmed cases per region during the time period of our study. Based on the confirmed cases count, the west region reported the highest COVID-19 cases, which matched the obtained results that the west was one of the least aware regions that scored a high percentage of negative sentiments. Whereas the second place of the cases counts came from the middle region, which shared almost the same level of awareness with the west region. In contrast, the north and south had the lowest number of COVID-19 confirmed cases, with 2% and 4%, respectively. This matches the currently obtained results of the level of awareness.

To sum up, the results of this study align with the announced report of the confirmed cases, which supports the study and the obtained results.

In conclusion, the developed model confirms its effectiveness at classifying Arabic Twitter data, to be used for prediction purposes based on sentiments. These results can help authorities in decision-making, regarding the appropriate actions based on the awareness level of each region. For example, since the south region showed a higher awareness level than others, the authorities may minimize restrictions in that area. In contrast, in the middle region, since individuals showed the lowest awareness level, the authorities may invest more time and methods to increase the awareness level in that region, or apply more restrictions to control the situation.

## 5. Conclusions

This research aimed to study Saudi Arabian citizens’ awareness levels of the COVID-19 pandemic and their attitudes toward pandemic control. This work assists decision-makers, to help them set suitable precautionary procedures for each region based on its level of awareness. The goals were achieved by collecting and preparing a dataset from the five regions of Saudi Arabia: north, south, east, west, and middle. Since Twitter is a very popular platform in Saudi Arabia, tweets were the main source of data in this research. Tweet datasets were collected separately for each region, according to location, based on specific keywords that were predefined as related to COVID-19 and precautionary procedures. The data were cleaned, preprocessed, and annotated into three categories: positive, negative, and neutral.

Three machine learning techniques were chosen for training the model. The results show that bigram TF-IDF with the SVM classifier produced the highest accuracy of 85%, which outperformed KNN and Naïve Bayes. The proposed model was used to predict the awareness of individuals per region; the south region showed the highest level of awareness at 65%, while the middle was the lowest among the regions.

For future work, we recommend applying feature selection and parameter tuning while building the machine learning models, and experiment using deep learning models. Moreover, we recommend taking into consideration the number of infected people per region to analyze the awareness.

## Figures and Tables

**Table 1:** Performance comparison of classifiers using N-gram feature selection.

N-Gram	ML Method	Accuracy	Precision	F-Score	Recall
**Unigram Counts**	KNN	0.57	0.53	0.52	0.52
	SVM	0.81	0.79	0.79	0.80
	NB	0.72	0.70	0.71	0.73
**Unigram TF-IDF**	KNN	0.62	0.55	0.53	0.53
	SVM	0.81	0.81	0.79	0.78
	NB	0.73	0.71	0.71	0.72
**Bigram Counts**	KNN	0.56	0.53	0.46	0.48
	SVM	0.82	0.81	0.81	0.81
	NB	0.78	0.77	0.77	0.77
**Bigram TF-IDF**	KNN	0.64	0.57	0.54	0.53
	SVM	0.85	0.87	0.84	0.82
	NB	0.80	0.78	0.78	0.78
